# Novel Host-Related Virulence Factors Are Encoded by Squirrelpox Virus, the Main Causative Agent of Epidemic Disease in Red Squirrels in the UK

**DOI:** 10.1371/journal.pone.0096439

**Published:** 2014-07-01

**Authors:** Alistair C. Darby, Colin J. McInnes, Karina Hansen Kjær, Ann R. Wood, Margaret Hughes, Pia Møller Martensen, Alan D. Radford, Neil Hall, Julian Chantrey

**Affiliations:** 1 University of Liverpool, Liverpool, United Kingdom; 2 Moredun Research Institute, Penicuik, United Kingdom; 3 University of Aarhus, Aarhus, Denmark; 4 King Abdulaziz University, Jeddah, Saudi Arabia; University of Texas HSC at San Antonio, United States of America

## Abstract

Squirrelpox virus (SQPV) shows little evidence for morbidity or mortality in North American grey squirrels (*Sciurus carolinensis*), in which the virus is endemic. However, more recently the virus has emerged to cause epidemics with high mortality in Eurasian red squirrels (*S. vulgar*is) in Great Britain, which are now threatened. Here we report the genome sequence of SQPV. Comparison with other *Poxviridae* revealed a core set of poxvirus genes, the phylogeny of which showed SQPV to be in a new Chordopoxvirus subfamily between the Molluscipoxviruses and Parapoxviruses. A number of SQPV genes were related to virulence, including three major histocomaptibility class I homologs, and one CD47 homolog. In addition, a novel potential virulence factor showing homology to mammalian oligoadenylate synthetase (OAS) was identified. This family of proteins normally causes activation of an endoribonuclease (RNaseL) within infected cells. The putative function of this novel SQPV protein was predicted *in silico*.

## Introduction

Poxviruses cause disease in many different taxa and have been described in insects, birds, reptiles and mammals [26]. The mammalian poxviruses are found both in terrestrial and marine environments with many restricted to one host species, but a number can cause disease in more than one species with some being zoonotic [22]. Squirrelpox virus (SQPV) has emerged in Great Britain as the cause of high mortality in Eurasian red squirrel (*Sciurus vulgaris*) populations whilst apparently leaving sympatric North American grey squirrel (*Sciurus carolinensis*) populations unaffected. The underlying mechanisms responsible for the pathogenicity of SQPV are not understood and hence the reason for the different disease manifestation in the two species cannot be fully investigated. In this paper we document the complete sequence of the SQPV genome (excluding the inverted terminal repeats), confirming that it is a species in the family *Poxviridae* and does not partition with any of the currently recognized genera.

### The disease and epidemiology

Red squirrels with squirrelpox show typical signs associated with poxvirus infections, with the formation of multifocal extensive ulcerative lesions around the mouth and eyelids [25]. Squirrelpox lesions contain high concentrations of virus which can be shed to the environment, possibly helping to sustain infection. Diseased individuals rarely survive infection and this has had a profoundly negative impact on the distribution of red squirrels in Great Britain [32], [34], [41]. Red squirrels only become infected where they are sympatric with the introduced North American grey squirrels which are considered to be the reservoir host of the virus. Although most grey squirrels in England and Wales appear to be serologically positive for antibodies to the virus, they rarely show signs of disease and indeed currently there are no known negative consequences of SQPV infection in grey squirrels [4], [33].

It had been postulated that competition for habitat and resources partially explains the replacement of reds by greys [42], however epidemiological modelling of squirrelpox suggests that grey squirrels replace red squirrel populations up to 25 times faster when the grey squirrel populations carry the poxvirus infection [30]. Current estimates put the remaining UK population of red squirrels at approximately 160,000 with 75% of those being in Scotland, predominantly in areas where there are either no grey squirrels or where reds are sympatric with grey squirrels which are serologically negative for SQPV. It has been suggested that the red squirrels will die out within 15–20 years if nothing is done to protect them from SQPV.

### Virulence genes and mechanisms

Viruses often encode proteins related to the immune system of their host in order to circumvent host immunity and improve viral survival. Poxvirus-encoded virulence factors target many different aspects of host immunity. The majority act to block or subvert the host anti-viral responses, and include proteins that manipulate apoptosis of host cells, inhibit the activity of both natural killer (NK) cells and cytotoxic T-cells and that bind and/or inhibit a multitude of host cytokines [1], [5], [16], [28], [35], [40].

The interferon (IFN) response system is targeted in many ways by poxvirus-encoded factors acting before and after IFN production [5]. Usually this involves binding and neutralization of IFN molecules [e.g. some poxviruses encode soluble IFN receptor homologues which prevent IFN action) or other proteins functioning in the IFN system [28]. Poxviruses also target specific enzymes in IFN-dependent pathways, such as protein kinase R [PKR). By targeting these enzymes the poxviruses help to prevent the shutdown of viral protein synthesis in the infected cell [10]. Outside of the poxvirus family there are other viruses that encode transcription suppressors of the IFN system (the HHV-8 virus encodes an IRF homolog which suppresses transcription) and viruses that encode RNA binding proteins preventing activation of PKR and oligoadenylate synthetase OAS)/RNaseL [17].

The emergence of SQPV in red squirrels raises important questions about the virus's evolution and it's host interactions. It is clear SQPV has the ability to transfer between host species and has coevolved mechanisms which allow attenuated pathogenesis and virulence within the grey squirrel resulting in asymptomatic infection, but producing a high degree of virulence in the red squirrel host.

Here we describe the genomic sequence of SQPV, discuss its phylogenetic relationship with the other *Poxviridae*, and describe novel virulence genes including one predicted to impede the host interferon response via interaction with the OAS system.

## Materials and Methods

### Ethics statement

The virus isolate used in this study was a from a necropsy sample taken from a red squirrel that had been found dead in the wild as previously described in [24]. The work was therefor not subject to animal welfare legislation in the UK and ethical permission for sampling was not sought.

### DNA Cloning and sequencing

Pox genomic DNA isolated directly from scabs from an infected squirrel [24] was cloned into SuperCos I cosmids (Stratgene, La Jolla, USA) [24]. Six cosmids with an average insert size of 35 kb were selected on the basis that they spanned the entire genome. DNA was prepared using the Plasmid Maxi Kit (Qiagen, UK) and pooled in equimolar proportions before being made into a single fragment shotgun sequencing library. Sequencing was conducted on a Genome Sequencer FLX System (Roche Diagnostics, UK) and assembled using Newbler (Roche Diagnostics, UK).

### Assembly finishing and annotation

After assembly the resulting contigs were filtered with BLAST to remove the non-viral sequences. The contigs were viewed in GAP4 (http://staden.sourceforge.net) and gaps closed by assembling Sanger sequences. Protein-coding genes were identified by GLIMMER [8], GenemarkS eukaryotic viruses model [23] and BLASTX using a viral protein database downloaded from NCBI FTP site (August 2009). Putative functions were inferred using BLAST against the NCBI databases and InterProScan [20]. Artemis v11 was used to organize data and facilitate annotation [31]. A full list of annotated genes is in Table S1.

### Phylogenetic analysis

The pox phylogeny was reconstructed using orthologous gene sets identified in other viral genomes using ORTHOMCL (default parameters), 39 orthologous gene clusters where identified across 19 pox taxa (Canarypox virus CNPV NC_005309; Fowlpox virus FWPV NC_002188; Sheeppox virus 17077-99 SPPV NC_004002; Goatpox virus Pellor GTPV NC_004003; Lumpy skin disease virus NI-2490 LSDV NC_003027; Rabbit fibroma virus RFV NC_001266; Myxoma virus MYXV NC_001132; Molluscum contagiosum virus MOCV NC_001731; Monkeypox virus Zaire-96-I-16g MPXV NC_003310; Camelpox virus CMLV NC_003391; Ectromelia virus ECTV NC_004105; Vaccinia virus VACV NC_006998; Cowpox virus CPXV NC_003663; Orf virus ORFV NC_005336; Bovine papular stomatitis virus BPSV NC_005337; Swinepox virus SWPV NC_003389), aligned with MUSCLE [9] and trimmed with GBLOCKS [39] Alignments of 40 genes (orthologous genes to SQPV013c, SQPV022, SQPV024c, SQPV026c, SQPV029, SQPV030, SQPV036c, SQPV038c, SQPV040c, SQPV041c, SQPV044c, SQPV046, SQPV048, SQPV049, SQPV050, SQPV052, SQPV055c, SQPV056, SQPV058, SQPV060, SQPV065c, SQPV067c, SQPV073c, SQPV075, SQPV080, SQPV088c, SQPV090c, SQPV092c, SQPV095, SQPV096c, SQPV099, SQPV100c, SQPV101c, SQPV104c, SQPV110c, SQPV113c, SQPV114, SQPV115, SQPV116, SQPV122c) were then concatenated and maximum likelihood trees calculated by JTT, estimated transition/transversion ratio, fix proportion of invariable sites were implemented using PHYML [15], 1000 boot replicates were performed.

### Structure modelling

Structural homologues were identified using predicted amino acid sequences of relevant ORFs submitted for analysis on the SWISS-MODEL database [2]. Estimated model reliability was calculated using QMEAN4 score (range 0–1, higher values indicate better fit). These data were displayed and manipulated in PYMOL (The PyMOL Molecular Graphics System, Version 1.3, Schrödinger, LLC).

## Results and Discussion

### Genome sequence

The *Poxviridae* are dsDNA viruses with linear genomes that range in size from ca. 135 kb to 360 kb. The final assembly for the SQPV genome (containing only one of the inverted terminal repeat [ITR] sequences) was within this range at 148 kb (one contig) with a GC content of 66.69% (Table 1). The previously available genomic data for SQPV [24] covered 60 kb from the right and left ends of the genome (∼40% genome), but lacked the central region of the sequence thought mainly to contain genes conserved across all poxvirus genera. The partial sequence of one of the two inverted terminal (ITR) sequences at the left hand end is included in the final assembly. The right hand ITR has the same sequence and size as the left hand ITR (3383 bp), but could not be resolved separately due to its identical sequence. There is one gene (SQPV_001) predicted to span the ITR/unique sequence boundary at the left end of the genome, but none at the right end of the genome, justifying the inclusion of only one copy of the ITR in the final assembly. The genome is therefore complete for all protein coding regions in the unique region, but lacks predicted extreme left and right hand ends of the genome, including the covalently closed terminal loops. It was estimated previously that the ITR was approximately 5 kb in length [24] and therefore the full genome size is predicted to be between 152–155 kb. The genome sequence has been submitted to the National Center for Biotechnology Information (NCBI) databases under the accession HE601899.

**Table 1:** Genome statistics.

Genome size, bp*	152,186
Size of assembly, bp**	148,803
Size without ITR, bp	145,420
Average sequence coverage	52x
Number of CDS	141
GC content percentage	66.69
CDS density, genes per kb	0.94
CDS average length, bp	1006
CDS coding percentage	94.7
Novel genes	26
Conserved pox/viral genes	115

* Both ITR regions included, but missing the extreme termini.

** only one ITR region.

**Table 2:** Summary of sequenced poxvirus genomes with abbreviations.

Genome	ICTV Abbreviation	Accession	Size (bp)	GC %	Group
Canarypox virus	CNPV	NC_005309	359,853	30.4	Avipoxvirus
Fowlpox virus	FWPV	NC_002188	288,539	30.9	Avipoxvirus
Sheeppox virus 17077-99	SPPV	NC_004002	149,955	25	Capripoxvirus
Goatpox virus Pellor	GTPV	NC_004003	149,599	25.3	Capripoxvirus
Lumpy skin disease virus NI-2490	LSDV	NC_003027	150,773	25.9	Capripoxvirus
Deerpox virus W-848-83	DPV	NC_006966	166,259	26.2	Cervidpoxvirus
Rabbit fibroma virus	RFV	NC_001266	159,857	39.5	Leporipoxvirus
Myxoma virus	MYXV	NC_001132	161,773	43.6	Leporipoxvirus
Molluscum contagiosum virus	MOCV	NC_001731	190,289	63.4	Molluscipoxvirus
Variola virus	VARV	NC_001611	185,578	32.7	Orthopoxvirus
Monkeypox virus Zaire-96-I-16g	MPXV	NC_003310	196,858	33.1	Orthopoxvirus
Camelpox virus	CMLV	NC_003391	205,719	33.2	Orthopoxvirus
Ectromelia virus	ECTV	NC_004105	209,771	33.2	Orthopoxvirus
Taterapox virus	TATV	NC_008291	198,050	33.3	Orthopoxvirus
Vaccinia virus	VACV	NC_006998	194,711	33.3	Orthopoxvirus
Cowpox virus	CPXV	NC_003663	224,499	33.4	Orthopoxvirus
Orf virus	ORFV	NC_005336	139,962	63.4	Parapoxvirus
Bovine papular stomatitis virus	BPSV	NC_005337	134,431	64.5	Parapoxvirus
Swinepox virus	SWPV	NC_003389	146,454	27.4	Suipoxvirus
Crocodilepox virus	CRV	NC_008030	190,054	61.9	unclassified Chordopoxvirinae
Squirrelpox virus	SPPV		148,445	66.7	unclassified Chordopoxvirinae
Yaba-like disease virus	YLDV	NC_002642	144,575	27	unclassified Yatapoxvirus
Tanapox virus	TANV	NC_009888	144,565	27	Yatapoxvirus
Yaba monkey tumor virus	YMTV	NC_005179	134,721	29.8	Yatapoxvirus

### Phylogentic position of squirrelpox virus

**Figure 1:**
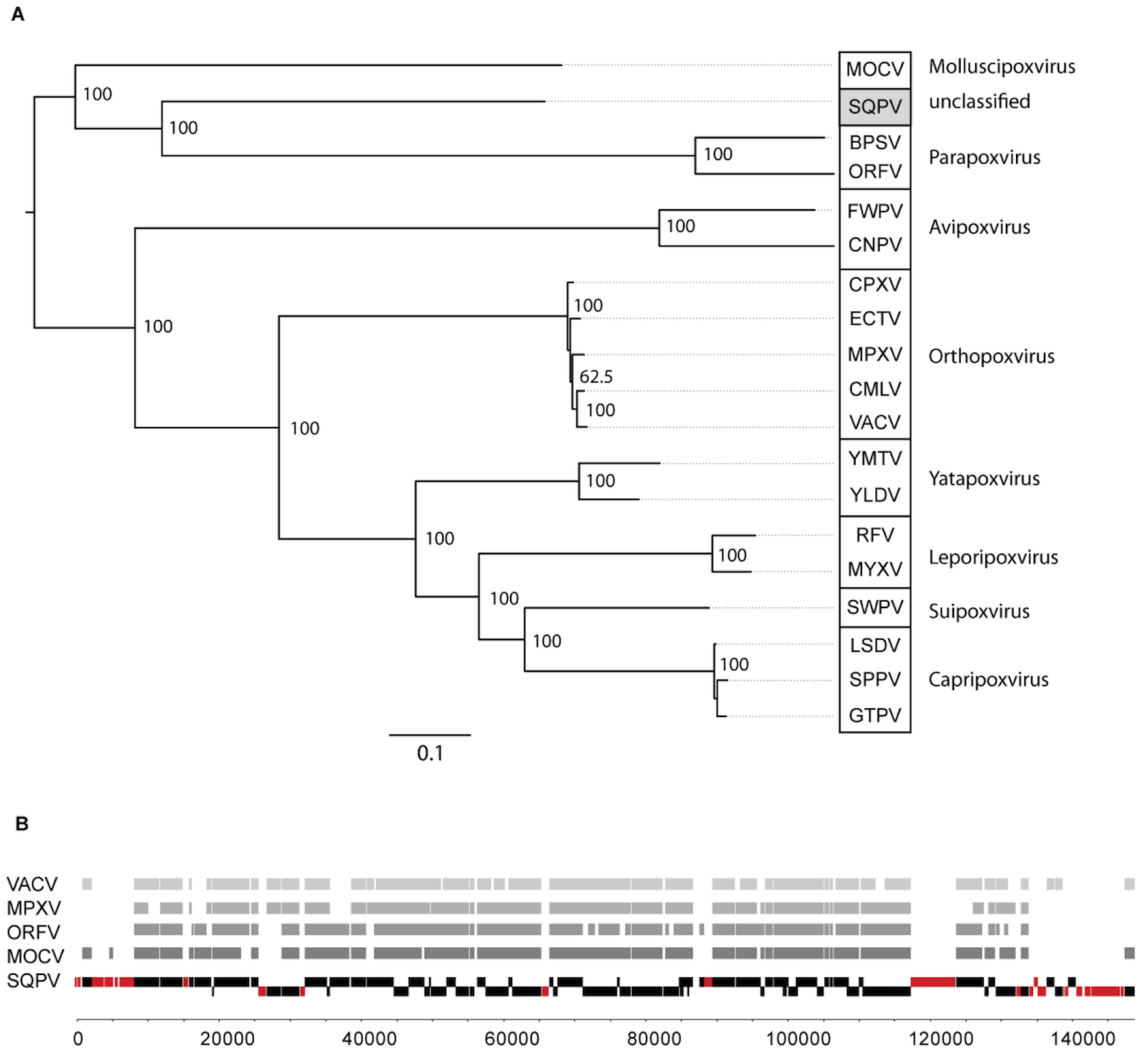
Squirrelpox virus (SQPV) phylogeny and genome structure. A. Poxvirus phylogeny showing the novel position of the SQPV. Midpoint rooted ML tree based on 39 conserved core genes from 19 taxa. Node values give percentage bootstrap support (n = 1000). See Table 2 for abbreviations. B. SQPV genome overview showing conserved poxvirus genes in grey and unique SQPV genes in red (displayed according to their coding strand). The grey bands show orthologous genes found in other poxviruses (displayed without coding strand information).

The phylogenetic analysis of the virus (Figure 1A) shows that squirrelpox virus is a member of the subfamily of chordopoxviruses. The tree topology and branch lengths suggest that SQPV shares a common ancestor with Molluscipoxvirus and the Parapoxviruses, but represents a distinct and novel genus. This was suggested previously [24] but this study provides a more robust phylogeny and is supported by multiple methods and individual gene trees (data not shown). The Phylogeny suggests a relatively close relationship with the *Parapoxvirinae* and the *Molluscipoxvirinae*, but the presence of the VACV-Cop F15 and D9 homologs in the SQPV genome, together with the positional conservation of the VACV-Cop F9 and F10 genes (cf. the parapoxviruses), differentiates SQPV from the parapoxviruses suggesting the viruses must have diverged before the gene rearrangements and losses occurred in the *Parapoxvirinae*.

### Comparative genomics

Chordopoxvirus genomes have a central core of conserved genes. This consists of a minimum (core) set of 88 genes which contains genes essential for virus viability and encode proteins involved in viral DNA replication, transcription and virion assembly [14]. These are all present in the SQPV genome (See Table S1) which also includes the extra two genes (VACV-Cop F15 and D9) that are reported to be found in all other chordopoxvirus genomes except the parapoxviruses [14]. In the central conserved core region of the SQPV genome there are an additional 15 genes, 10 of which appear to be unique to SQPV. These 10 genes include two MHC class I-like molecules, an orthologue of 2′,5′-oligoadenylate synthetase (see below) and a likely member of the viral A-type inclusion protein superfamily. The remaining six have no known or predicted function. Eighty-two percent [115/141) of the genes in the genome have homologs in another pox genome, (Table S1). Most of the conserved genes are located in the middle of the genome (Figure 1B), whilst the more variable and novel genes that are likely to define host range and virulence (many of which have been shown by others to be non-essential for *in vitro* replication), are located at either end of the genome. Our data corrects mistaken annotation in the original description of these flanking regions [24].

Unlike the parapoxviruses and the *molluscum contagiosum virus*, SQPV contains a thymidine kinase (TK) gene in the central region of the genome similar to the majority of other poxviruses. The SQPV TK gene, however, is not positionally conserved, in comparison to other poxviruses, and BLAST database searching suggests its closest orthologue is from the rat (*Rattus norvegicus*) and other rodents. This might suggest that the SQPV TK gene is a relatively recent, independent, acquisition and further analysis of its sequence may help in determining some of the evolutionary history of the virus.

### Putative immune modifiers

#### Major histocompatability complex (MHC) Class I

Many viruses have evolved ways to interfere with natural killer (NK) cell activation [27] through encoding MHC Class I homologs. This allows the virus to 1) down-regulate host MHC expression and 2) avoid NK surveillance through surface expression of viral MHC homologs. Such viral MHC proteins are often encoded in herpesviruses, but rare in poxvirus genomes. The SQPV genome is unusual amongst poxviruses, encoding three putative MHC-like homologs (SQPV_004, SQPV_027 and SQPV_064), spread throughout the genome. SQPV_004 is most conserved with class I (127/291(44%) identity to human MHC class 1, AAA57146), whilst SQPV_027 and SQPV_064 are much less conserved (72/273 (26%) and 47/194 (24%) respectively) (Figure S1). The three genes share little identity to each other (SQPV_004 to SQPV_027 – 24%: 65/271 aa. SQPV_004 to SQPV_064 – 20%: 11/55 aa. SQPV_027 to SQPV_064 – 22%: 15/68 aa), which may suggest they represent independent acquisition events or arose as the result of an ancient duplication before subsequent divergence. Both SQPV_004 and SQPV_027 are predicted to have similar transmembrane domains to class I molecules, but SQPV_064 appears to lack a C terminal hydrophobic domain, suggesting it could be secreted. The poxvirus *Molluscum contagiosum virus* (MOCV) encodes one MHC class I homolog (MOCV080R gene product) [36]. This protein is largely sequestered in the endoplasmic reticulum and Golgi, is capable of binding β2-microglobulin and is not detected on the cell surface. It's role in virulence and whether or not it is involved in host NK-cell avoidance is not understood. Further studies are needed on the cellular location of the SQPV MHC molecules and to clarify their possible role in avoiding NK-cell surveillance and immune evasion.

#### CD47 and anti-apoptosis

The SQPV CD47 homolog shares 25–29% amino acid identity with a range of mammalian CD47 molecules and homology to the A38L protein found in Vaccinia virus (strain Copenhagen; VACV_Cop) and a range of other poxviruses. It shares strong sequence similarity to the immunoglobulin superfamily domain of CD47 [18] (Figure S2; QMEANscore4 0.51) and is predicted to be membrane bound. Key cysteine residues associated with disulphide bond formation appear conserved whereas other residues critical for signal regulatory protein (SIRP) interaction are altered suggesting that SQPV CD47 may be structurally intact but fail to activate CD47 receptors.

In mammals, CD47 is widely expressed and its function is complex. It has been implicated in a range of T-cell regulatory functions including both inhibiting [12], and promoting [29] apoptosis, depending on its local molecular interactions. Deletion of the myxoma virus CD47 homolog (M128L) demonstrated that this gene is a virulence factor, necessary for lethal infections in susceptible rabbits, while it was fully dispensable for virus replication *in vitro* [6]. Animals infected with the M128L-deleted virus showed greater activation of monocyte/macrophage cells in infected and/or lymphoid tissues.

#### Interferon (IFN)

The interferon system is activated in response to viral infection and plays an important role in the host defence system. Interferons released into the extracellular space bind interferon receptors on neighboring cells to induce an alert system. The receptors activate the JAK-STAT pathway and induce a range of interferon stimulated genes. Various poxviruses encode proteins that either bind (or are predicted to bind) interleukin-18 (IL-18), an inducer of type II IFN, or are mimics of both type I and type II IFN receptors [38]. In the case of viral mimics they are thought to work by competitive inhibition, preventing the IFNs from binding to their natural receptors [35]. Vaccinia virus (VACV) is also known to be able to inhibit the IFN-induced activation of STAT-1 [16] whilst it is thought that MOCV may be able to inhibit IFN-induced NF-κB activation [13]. SQPV does not seem to encode such genes but does encode a putative PKR inhibitor and, uniquely amongst the poxviruses, a 2′-5′ oligoadenylate synthetase (OAS)-like protein.

#### Protein Kinase (PKR)

**Figure 2:**
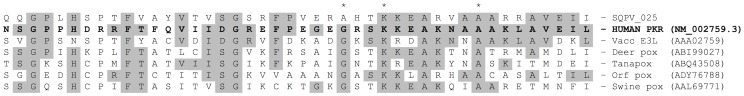
Comparison of the dsRNA interaction domain of SQPV_025, VACV_Cop E3L, human protein kinase R (PKR) and related proteins from other poxviruses. Conserved residues with the human form are indicated by a grey background. Asterisks above the alignment indicate those residues that have been shown to be necessary for dsRNA binding in E3L [7].

Double-stranded RNA-activated protein kinase is a key component mediating the antiviral actions of IFN that restricts viral replication by phosphorylating eukaryotic initiation factor-2a-subunit, an initiator of protein synthesis, reducing levels of viral protein [10]. Many viruses have evolved mechanisms to down regulate PKR function [11]. The SQPV_025 is homologous to VACV_Cop E3L (41% identity over 69 amino acids toward N terminus), a host range gene, which is required for VACV pathogenesis [3]. SQPV_025, VACV_Cop E3L and other poxvirus homologs share a small region of similarity in their carboxy terminus to an N-terminal domain of PKR (Figure 2). No other significant areas of homology exist between PKR and these poxvirus proteins. In vaccinia, this region has been shown to be responsible for inhibiting the activation of PKR both by binding to and sequestering dsRNA molecules [7], and by direct interaction with PKR itself [37].

#### Oligoadenylate synthetase (OAS)

**Figure 3:**
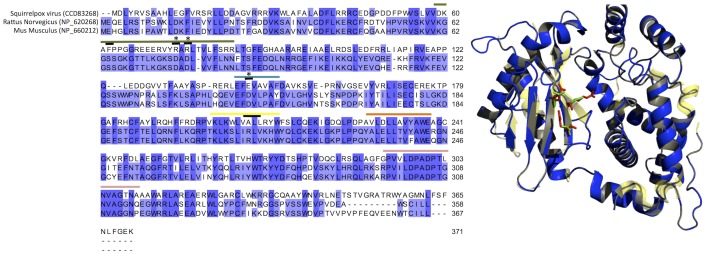
A. Amino acid alignment of mouse and rat 2′-5′ oligoadenylate synthetase with the putative squirrel poxvirus homolog. Coloured lines above the alignment indicates conserved functional regions and black lines indicates critical residues for function. Colours indicate different functional domains (Green, P-loop; Blue, PAFDAL; Yellow, LIRL; Peach, YALELLT and Pink, RPVILDPADP). The P-loop and PAFDAL domains hold the aspartic triad indicated by asterisks. The blue shading of the residues indicates the level of conservation, Dark blue is conserved in all species while light blue is conserved in just two. B. SQPV_0850 2′-5′ oligoadenylate synthetase – like molecule (blue) mapped to porcine 2′-5′ oligoadenylate synthetase OAS1 (PDB ID:1px5) (light yellow shadow); Green residues indicate the active DDD triad. Red residues indicate the equivalent residues RRE in the SQPV-OAS1 structure.

OAS proteins are activated by dsRNA binding, leading to the OAS-catalysed synthesis of 2′-5′oligoadenylates (ppp(A2′p)*n*5′A, abbreviated to 2-5A) from ATP. RNase L, a latent endoribonuclease, becomes activated through binding 2-5As and degrades both viral and cellular RNA, including cellular rRNA [19]. This antiviral response results in inhibition of protein synthesis and in some cases apoptosis. Protein alignments (Figure 3) show that the SQPV-OAS1 (SQPV_085) has a similar sequence to mammalian OAS1 proteins (128 identities out of 348 (37%) to porcine OAS [NP_999468]). The primary characteristics of the mammalian enzymes are: 2-5A synthesis, dsRNA binding, binding of other OAS enzymes, binding of 2-5As and ATP.

By *in silico* analysis, the SQPV-OAS1 gene is not conserved at amino acid residues known to be essential for normal OAS activity, lacking important aspartic triad residues [21] (Figure 3). A prediction of the tertiary structure based on the porcine OAS1 crystal structure (PDB ID:1px5) is shown in Figure 3b (QMEAN4 score 0.53). These observations suggested that SQPV-OAS1 protein should be capable of binding dsRNA, but is not expected to catalyse 2-5A synthesis. By doing so it may be that this viral protein is able to prevent or at least reduce the activation of RNase L.

## Conclusions

It is clear from these results that SQPV is both in a novel taxonomic position in the chordopoxviruse subfamily which indicates a possible new genus of poxvirus, and that this virus has a previously undescribed diversity of virulence genes encoded in its genome. It would seem that this virus has adapted to it's host niche using genes commonly associated with pox virulence (e.g. CD47, PKR), but has also acquired novel genes such as multiple MHC molecules, and a way of potentially inhibiting the 2-5A system of the host.

The precise advantage and role of the specific virulence genes, and how they contribute to the difference in disease between grey squirrel populations in which it appears non-pathogenic and red squirrel populations in which it is emerging as lethal infection, is still to be determined.

## Supporting Information

Figure S1 **Structural alignment of A. SQPV_004 (blue) aligned to class I MHC H-2Kk (light yellow) and β 2-microblobulin (red) with a peptide (green) in the peptide binding groove (based on PDB entry DOI:10.2210/pdb1zt7/pdb).** Structural alignment of B. SQPV_027 (blue) aligned to class I MHC-like HFE (yellow) and β 2-microblobulin (red) (based on PDB entry DOI:10.2210/pdb1a6z/pdb) Structural alignment of C. SQPV_064 (blue) aligned to class I MHClike HFE (yellow) and β2-microblobulin (red) (based on PDB entry DOI:10.2210/pdb1a6z/pdb).
(PDF)

Figure S2 **A. Predicted structure of SQPV_132 (VACV_Cop A38L) (Blue) with the extracellular domain of of CD47 (light yellow) and SIRPα (green) (based on PDB entry 10.2210/pdb2jjs/pdb).** B. Alignment of SQPV_132 with CD47 antigen isoform 3 precursor [Homo sapiens] (NM_001025079.1. The cysteine residues that form a conserved disulphide bond between β sheets [18] are highlighted in yellow. Residues mediating specific polar interactions with SIRPa are in magenta.
(PDF)

Table S1 **Gene table function and vaccinia virus homology.** The table shows the location and annotation of the predicted SQPV genes plus the predicted ortholog in other species. The “Nearest Poxvirus GeneID” column the ID of the gene with the top blast hit. The Product column gives the annotation of the predicted product based on the best blast hit.
(XLSX)
