# Supplementary figures and images for: Novel Host-Related Virulence Factors Are Encoded by Squirrelpox Virus, the Main Causative Agent of Epidemic Disease in Red Squirrels in the UK
Source: PLoS One. 2014 Jul 1;9(7):e96439. doi: 10.1371/journal.pone.0096439 (PMC4077651; doi:10.1371/journal.pone.0096439)

Figure S1

A)

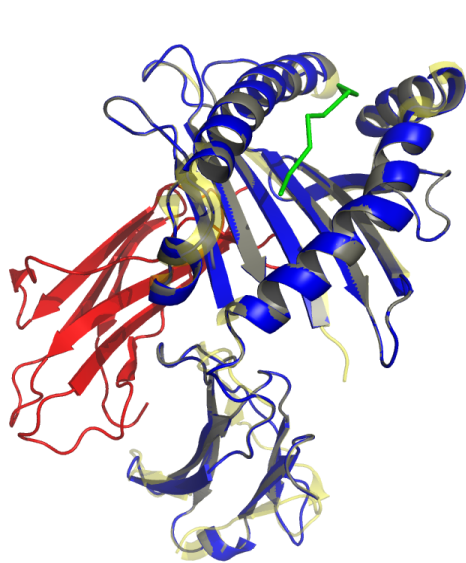

B)

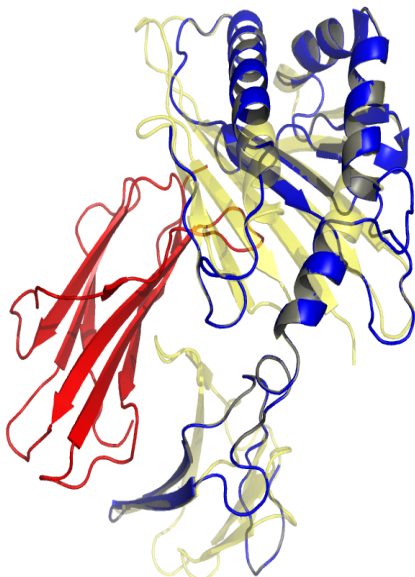

C)

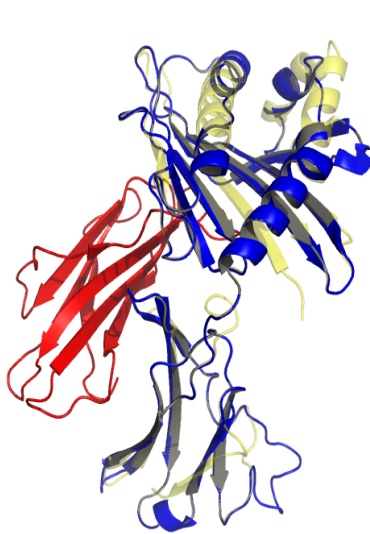

Supplement: Figure S1 — Structural alignment of A. SQPV_004 (blue) aligned to class I MHC H-2Kk (light yellow) and β 2-microblobulin (red) with a peptide (green) in the peptide binding groove (based on PDB entry DOI:10.2210/pdb1zt7/pdb). Structural alignment of B. SQPV_027 (blue) aligned to class I MHC-like HFE (yellow) and β 2-microblobulin (red) (based on PDB entry DOI:10.2210/pdb1a6z/pdb) Structural alignment of C. SQPV_064 (blue) aligned to class I MHClike HFE (yellow) and β2-microblobulin (red) (based on PDB entry DOI:10.2210/pdb1a6z/pdb). (PDF) [file pone.0096439.s001.pdf]

Figure S2

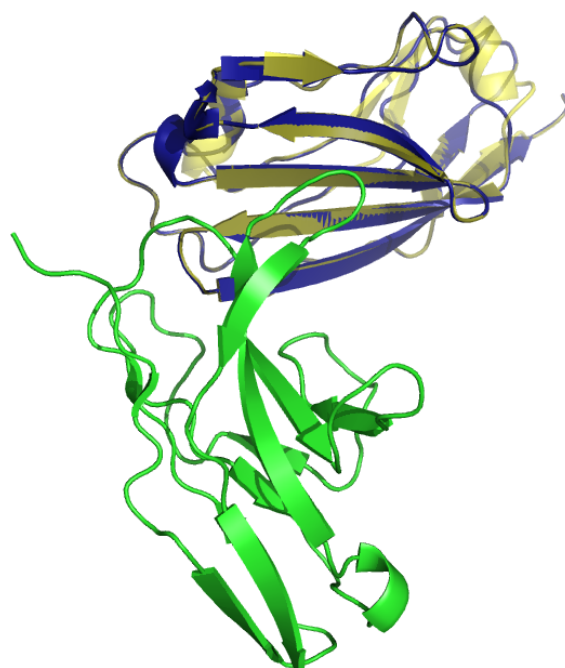

Supplement: Figure S2 — A. Predicted structure of SQPV_132 (VACV_Cop A38L) (Blue) with the extracellular domain of of CD47 (light yellow) and SIRPα (green) (based on PDB entry 10.2210/pdb2jjs/pdb). B. Alignment of SQPV_132 with CD47 antigen isoform 3 precursor [Homo sapiens] (NM_001025079.1. The cysteine residues that form a conserved disulphide bond between β sheets [18] are highlighted in yellow. Residues mediating specific polar interactions with SIRPa are in magenta. (PDF) [file pone.0096439.s002.pdf]
